# Managing Carbon Sinks in Rubber (*Hevea brasilensis*) Plantation by Changing Rotation length in SW China

**DOI:** 10.1371/journal.pone.0115234

**Published:** 2014-12-23

**Authors:** Syed Moazzam Nizami, Zhang Yiping, Sha Liqing, Wei Zhao, Xiang Zhang

**Affiliations:** 1 Key Laboratory of Tropical Forest Ecology, Xishuangbanna Tropical Botanical Garden, CAS Menglun Yunnan 666303, China; 2 University of Chinese Academy of Sciences, Beijing 100049, China; 3 Faculty of Forestry, Range Management & Wildlife, Arid Agriculture University Rawalpindi 46300, Pakistan; Institute of Tibetan Plateau Research, China

## Abstract

Extension of the rotation length in forest management has been highlighted in Article 3.4 of the Kyoto Protocol to help the countries in their commitments for reduction in greenhouse gas emissions. CO_2_FIX Model Ver.3.2 was used to examine the dynamics of carbon stocks (C stocks) in a rubber plantation in South Western China with the changing rotation lengths. To estimate the efficiency of increasing the rotation length as an Article 3.4 activity, study predicted that the rubber production and C stocks of the ecosystem increased with the increasing rotation (25, 30, 35, 40 and 45 years). While comparing the pace of growth both in economical (rubber production) and ecological (C stocks) terms in each rotation, 40 years rotation length showed maximum production and C stocks. After elongation of 40 year rotation to four consecutive cycles, it was concluded that the total C stocks of the ecosystem were 186.65 Mg ha^-1^. The longer rotation lengths showed comparatively increased C stocks in below ground C stock after consecutive four rotations. The pace of C input (Mg C ha^-1^yr^-1^) and rubber production indicated that 40years rotation is best suited for rubber plantation. The study has developed carbon mitigation based on four rotation scenarios. The possible stimulated increase in C stocks of the entire ecosystem after consecutive long rotations indicated that the emphasis must be paid on deciding the rotation of rubber plantation in SW China for reporting under article 3.4 of the Kyoto Protocol.

## Introduction

Forest plantations have been considered to measure carbon sequestered from the atmosphere and mitigate future climate change [1]. Globally, tree plantations cover 396108 ha in 2005, and still increasing with a relatively annual expansion rate of 2%. While reforestation on the natural forest land accounts for about half of the overall increased area of tree plantations [2]. In a meta-analysis [3], the forest plantation has a 28% lower C storage compared to natural wood. This led to a doubt against the replacement of natural forests by the plantations as a measure of climate change mitigation. Nevertheless, most of the plantation forests at current rotation length do not achieve their maximum biological storage yet; prolongation of the rotation period generally results in increased C sequestration [4]. Additionally, there is also the possibility that plantation forests of very old ages would go forward to accumulate C, since recent surveys have reported that old-growth natural forests could all the same operate as a C sink [5], [6]. However, over mature or old-growth plantation forests are rarely included in comparison with natural forests so far. For instance, the mean stand age of plantation forests in the synthesis of [3] was just 27 years. Therefore, it is fair that the reduced sequestration potential of plantation forests was merely due to their current rotations too short for C pool to regain to the pre-disturbance level. However, there is still a lack of explicit trajectories of post-harvest C stocks in tree plantations, particularly those established on the natural forest lands, which contributes to the uncertainty about the role of plantation forests in global terrestrial ecosystem C cycle [7].

Traditional methods only utilize the forest resource inventory information for statistical analysis, and researches on forest C storage mainly focus on biomass and soil [8]. With the cross-integration between the diverse disciplines, computer simulation has been widely applied in several areas of environmental science. The computer model can integrate all aspects and the limiting components to calculate the C sequestration potential combined with the inventory information, providing a novel and viable means for forest ecosystem C sequestration potential analysis [9].

Rotation length (planned time of the establishment of a forest stands to its final felling) is considered to be an effective forest management activity for controlling the C stocks in forests [10], [11], [12], [13]. It affects the C stocks of both trees and soil and, through the effects on the quantity and the quality of harvested timber. For the application under Article 3.4 of the Kyoto Protocol a change in rotation length is also seen as a forest management activity that countries may choose to help them meet their commitments for reduction of greenhouse gas emissions [14], [15], [16].

The C stocks of trees increases with increasing rotation length, but the C stocks of soil do not necessarily [11]. By comparing, the effects on soil organic C (SOC) remain unclear yet [17]. Although the increased pattern occurred in about one-fifth of the individual studies, a statistically insignificant trend predominated for soil C dynamic over age sequence [18]. Any reduction in the soil C stock would, in addition, make it necessary to assess alterations in the C stocks of forests for Article 3.4 the Kyoto Protocol. A country may decide not to account for one or more of the five C stocks named, i.e. Aboveground biomass, belowground biomass, litter, dead wood, and soil organic matter, only if it can show that the stock is not decreasing [15]. Model simulations of different forests help in exploring the effects of rotation length on the C stocks of woods. Estimates of the rotation length effects on the C stocks of the natural forests and plantations are scarce, especially those that account for the dynamics between the different stocks of forest carbon. This lack of knowledge is exemplified, for instance, in the estimates made in the special report of the Intergovernmental Panel on Climate Change on Land Use, Land-Use Change and Forestry [16]. The numbers given there, accounts only for biomass and are founded on a simple assumption that a 15% increase in rotation length increases biomass by 5%. The large scale of afforestation/reforestation activities during the last three decades has made China the greatest acreage with plantations, constituting about one-third of the global plantation area [19]. To assess the implications of altering the rotation length of forest carbon, more thorough analyses are needed in this regard. Therefore, the present study was carried out to assess the potential of C sequestration and rubber production in the existing rubber plantation (*Hevea brasilensis*) of South Western, China. Based on the forest inventory data and literature, using a CO_2_FIX model a reference for more comprehensive and accurate estimate at different rotation lengths has been determined. The specific aims of the study were (i) to assess the effects of rotation length on above and below ground C as well as on rubber production and (2) to determine the rotation length where the highest carbon sink eligible under Article 3.4 of the Kyoto Protocol .

## Materials and Methods

### Study site

The rubber plantations situated in Xishuangbanna Tropical Botanical Garden (XTBG, 21^o^41′N, 101^o^25′E), locates at the northerly edge of the Asian tropical zone at an elevation of 570 m ASL were investigated in this study. The annual mean temperature is 22°C and the rainfall is 1496 mm. The climate is typically seasonal, which is dominated by the tropical southern monsoon from the Indian Ocean during May–October and by subtropical jet streams during November–April. Consequently, three seasons are exhibited which were defined by former studies in this region [20]. They are a cool-dry season (November–February), hot-dry season (March–April) and rainy season (May–October). The cool-dry season is the coolest period with a mean temperature of 17°C. There is dense fog in the morning, but hardly any rainfall (average 29 mm mo^−1^). The following hot-dry season is a transitional period with more rainfall (average 61 mm mo^−1^) and higher temperature (average 22°C). In the rainy season, rainfall comprises about 84% of the annual amount and mean temperature is the highest (25°C).

### The CO_2_FIX V3.2 Model

The CO_2_FIX V3.2 is an ecosystem level simulation model that quantifies the C stocks and fluxes in the forest using the so-called full carbon accounting approach, i.e. Calculating changes in carbon stocks in all carbon pools over time [21]. The total carbon physically stored in the system at any time (C_Tt_; Mg C ha^−1^) is considered to be 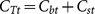



Where C_bt_ is the total carbon stored in living (above plus belowground) biomass at any time ‘t’ (Mg ha^−1^) and C_st_ is the carbon stored in soil organic matter (MgC ha^−1^).

### Carbon stored in living biomass

Biomass module considered C stocks per unit area of the biomass, as affected by the growth of the stem (including bark), foliage, branches, roots, and the mortality of the vegetations and logging. The increase of the branches, foliage and root biomass are determined by the coefficient of the relative proportion compared with the increase of the stem biomass. The carbon stored in living biomass (Cb_t_) of the whole forest stands, can be expressed as the sum of the biomasses of each cohort: 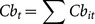



Where 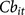
is the carbon stored in the living biomass of cohort ‘i’ at time ‘t’ (MgC ha^−1^).
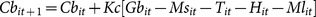



Where “Cb_it_” is calculated as the balance between the original biomass, Gb_it_ is biomass growth, T _it_ is the turnover of branches, foliage and roots, Ms_it_ is tree mortality due to senescence, H_it_ is the harvest and Ml_it_ is mortality due to logging. Kc is a constant to convert biomass to carbon content (Mg C per Mg biomass dry weight).

### Carbon stored in soil organic matter

Soil C stocks can be estimated by the dynamic soil module of YASSO in the model. The input component of soil C could be directly imported from biomass module. The module comprised three residual portions and five decomposing parts.

To gauge the effect of the changing rotation length from the currently (30–35 years) used, we studied differences in the final C stocks in the ecosystem (both above and below ground) at the completion of the first cycle and consecutive four cycles of that particular rotation (25, 35, 40 and 45years). The simulation time in the study was considered as four consecutive cycles for each rotation (25, 30, 35, 40 and 45years). Total change in C stocks of the trees and soil was determined by the difference between the final C stocks of the first cycle and fourth cycle in a particular rotation (25, 30, 35, 40 and 45years). We refer readers to [22] and [23] for a complete description of the CO_2_FIX model.

### Model parameters

#### Area characteristics

The area characteristics showed in Table 1 was used for simulation of C stocks through CO_2_FIX. The basic parameters used for simulation of the C stocks by the model are stem volume growth and allocation pattern to the other tree compartments (foliage, branches and roots). Carbon stocks in living biomass are calculated as the balance between growth on the one hand and turnover, mortality and harvest on the other hand. Litter from turnover and mortality processes and logging slash form the input for the soil module.

**Table 1:** Characteristics of the study plots.

S.No	Cohort	DBH*(cm)	CAI*(m^3^ ha^−1^ yr^−1^)	Plant Density (Trees ha^−1^)
1	1(2 years)	4.8+0.31	1.90	430
2	2 (6 years)	12.8+0.49	6.80	430
3	3 (9 years)	16.7+0.24	10.14	430
4	4 (11 years)	18.9+0.91	12.94	430
5	5 (13 years)	19.2+1.03	11.49	430
6	6 (16 years)	22.4+1.09	11.85	430
7	7(36 years)	37.5+1.68	28.58	430

* DBH: Diameter at breast height, CAI: Current Annual Increment.

#### Biomass module

The input data for biomass stimulation include current annual increment (CAI) of the stem wood volume (m^3^ ha^−1^ yr^−1^), biomass turnover rate, initial biomass, growth and mortality of each functional group relative to standing biomass, and interactions within and between the functional group. The data regarding diameter at breast height, height (to calculate volume and CAI), biomass growth and mortality of trees was collected from the rubber plantation of different ages (established in Xishuangbanna). Generic turnover rate of branches, foliage and roots were taken from [24]. Wood density (dry) of rubber (0.53 Mg m^−3^) was obtained from [25]. Comparison of dbh and height of the trees at a particular age represented promising results with [26]. The volume calculations were based on diameter and height parameters [27]. The reason for comparing with the [26] is the ground truths taken from plantation, while the growth and yield tables were not considered as they are often made in fully stocked stands and the plantations are established on some fixed spacing. Biomass regression equations were employed in our calculations for estimating the biomass of the tree components (branches, foliage and stems). When the stem biomass were estimated using basic wood density of the rubber, the result closely resembles the estimates of [26] models, so in the present study their models for estimation of biomass of branches, foliage and roots were observed.

#### Soil module

Parameterization of soil module for soil carbon requires litter input (MgC ha^−1^ yr^−1^) from foliage, fine roots, branches, coarse roots and stems, quantified from turnover rates, natural mortality, management mortality, and logging slash provided by the simulator in other modules of the model. Mean temperature and rainfall for the region is required for computation of potential evapotranspiration for the region, important in determining rates of decline. The size of non-woody litter, finer and coarse litter pools is determined by inputs from diverse sources of litter, minus the fractionation rate per pool. The proportion allocated to soluble compounds, holocellulose, and lignin-like compounds is in turn determined by fractionation rates and litter quality classes [22]. The baseline situation for simulation in the current study is shown in Table 2.

**Table 2:** Some parameters used by CO_2_FIX Model.

Turnover rates (1/yr.)			Growing Season PET (mm)	Allocation of Biomass (fractions)						
Foliage	Branches	Roots		Stem Log wood	Stem Slash	Branch Log wood	Branch Slash	Foliage Slash	Slash Fire wood	Slash Soil
0.50	0.50	0.50	1143.19	0.95	0.05	0.75	0.25	1.0	0.05	0.95

Initial Soil Carbon Estimates (MgC ha^−1^) (YASSO model parameters)						
NWL	FWL	CWL	SOL	HCL	LC	Humus
1.25	0.87	3.06	0.49	1.12	2.18	4.00

PET  =  Potential Evapotranspiration, NWL  =  Non woody Litter, FL  =  Fine woody Litter, CL  =  Coarse woody Litter, SOL  =  Soluble compounds, HCL  =  Holocellulose. LC  =  Lignin.

## Results

### C stocks of the ecosystem

**Figure 1:**
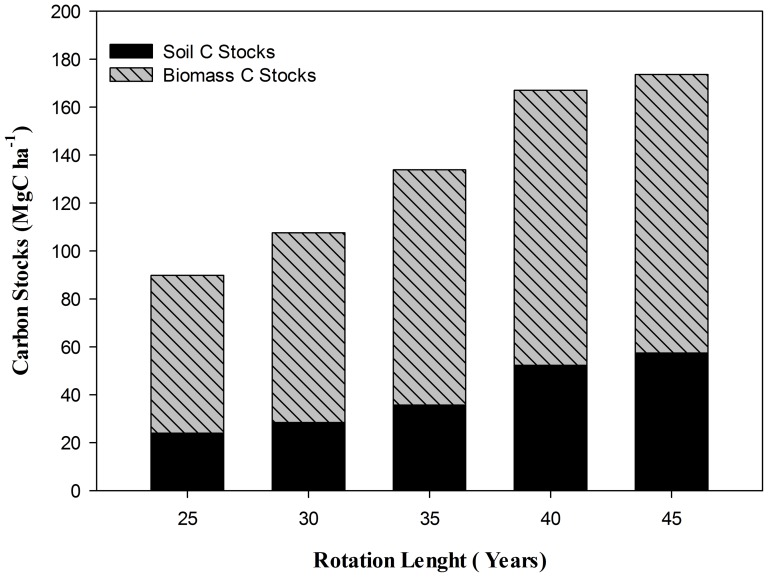
Total C stocks (MgC ha^−1^) at different rotation lengths (Year).

To demonstrate the mechanism that determines the effect of rotation length on the C stocks, five different rotations (25, 30, 35, 40 and 45 years) in rubber plantation were considered. The total C stocks (MgC ha^−1^) were larger the longer was the rotation length. This is because the time of production increased with the increasing rotation length. The total C stocks differed significantly across all the rotation lengths with the highest (173.60 MgC ha^−1^) at rotation length of 45 years and least (89.86 MgC ha^−1^) in 25 years rotation length (Fig. 1). When the percentage contribution in C stocks of the ecosystem both in above and below ground pools was determined, it was found that at the rotation length of 25 years the total C stocks (MgC ha^−1^) equals to 89.86 in which below ground and above ground biomass contributes 26.66% or (23.94 MgC ha^−1^) and 73.34% or (65.91 MgC ha^−1^). Likewise, at the end of the other rotations (30,35, 40 and 45 years) the contributions in percentage of above ground biomass were 73.64% or (79.21 MgC ha^−1^), 73.36% or (98.21 MgC ha^−1^), 67.53% or (114.79 MgC ha^−1^), and 66.90% or (116.15 MgC ha^−1^) respectively.

Likewise, the percentage contribution from below ground biomass also varied significantly with the changing rotation length. The highest c stocks and % contribution of belowground biomass was observed in the highest rotation length, viz a viz 45 years and was equal to 33.10% or 57.45 MgC ha^−1^. The study revealed that percentage contribution of the below ground biomass in total C stocks at short rotations (25, 30 and 35 years) remained non significant, but it becomes significantly varied at longer rotations of 40 and 45 years (32.47 and 33.10%; Fig. 1). Similarly, the contribution of above ground biomass was higher than below ground biomass at shorter rotation lengths as compared to longer rotation lengths.

### Rate of C input in above and below ground biomass

**Figure 2:**
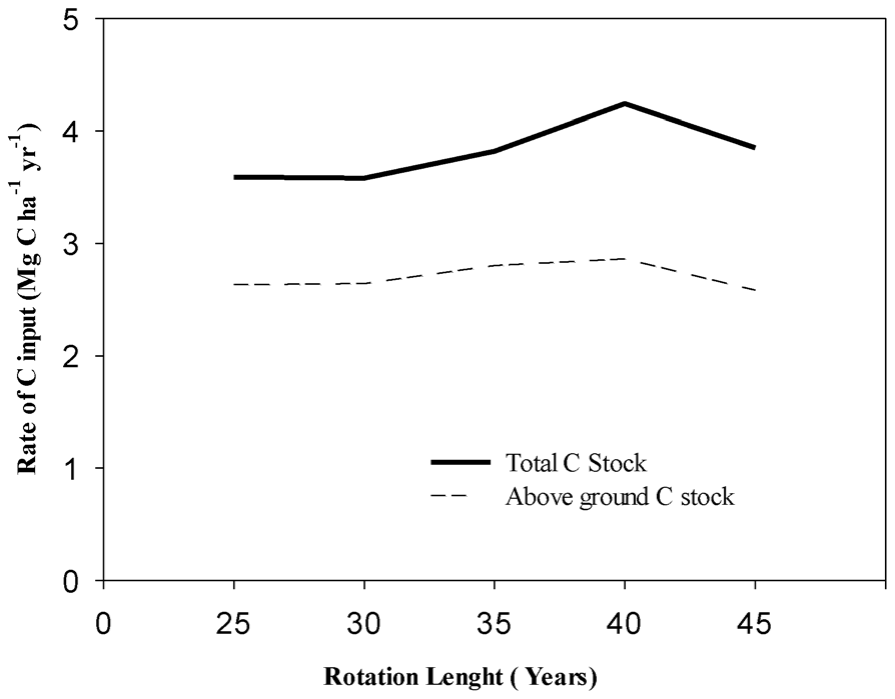
Rate of C input (Mg C ha^−1^ yr^−1^) in total C stock at different rotation lengths.

The study revealed that the C stocks of entire ecosystem increased with longer the rotation length (Fig. 2). The rate of C input (Mg C ha^−1^ yr^−1^) showed by the each rotation length (25, 30, 35, 40 and 45) was 2.63, 2.64, 2.80, 2.86 and 2.58 respectively. The 40 years rotation length showed the maximum rate of C input (2.86 Mg C ha^−1^ yr^−1^) in above ground biomass. This is because the current annual increment (CAI) and mean annual increment (MAI) reaches the apex period at the age of 40 years and after that the CAI starts declining. It is the point where the CAI and MAI culminates and gives highest productivity. In forestry the point where the CAI and MAI graph cross each other and then CAI starts declining is considered as a rotation determination point. The study revealed that at the age of 40 years the rate of input of C stock (Mg C ha^−1^ yr^−1^) remained highest and then it starts declining. So 40 years rotation is best for rubber grown in Xishuangbanna SW China in the context of the maximum C sink.

### Simulations of C stocks for four consecutive cycles in extended rotations

**Figure 3:**
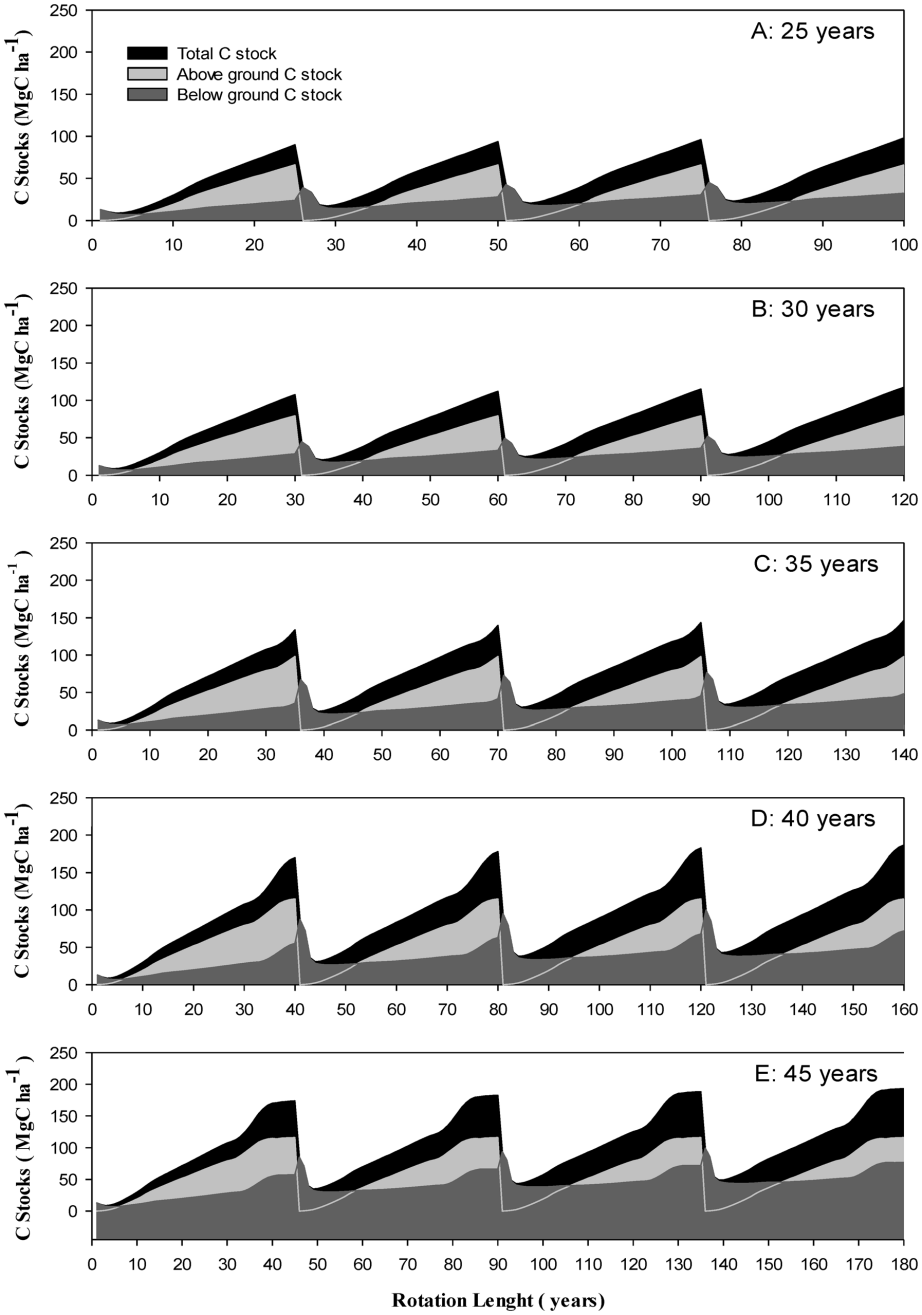
Simulation of C stocks (Total C stocks, above and below ground C stocks) for consecutive four cycles at different rotation lengths in Rubber Plantation.

The study also determined the C stocks dynamics in four consecutive cycles of different rotation by running simulations. The total C stocks (MgC ha^−1^) at the end of 1^st^ rotation (first 25 years) in case of 25 years rotation length was 89.86 Mg ha^−1^. The simulation results showed that at the end of four consecutive rotations of 25 years i-e 100 years, these stocks changed to 98.03 MgC ha^−1^. Similarly the other rotation length also increased the stock with the significantly different growth rate (Fig. 3).

### Economics and ecology of the rubber plantation

**Figure 4:**
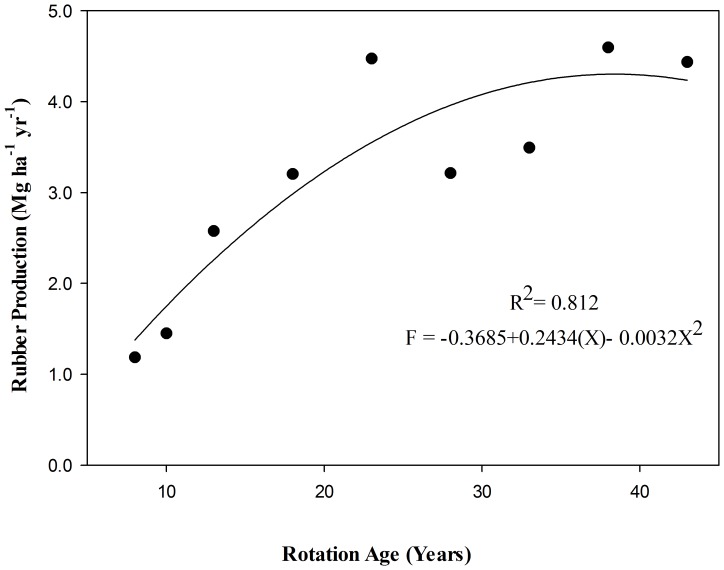
Regression Analysis of Rotation Lengths (Years) and Rubber Production (Mg ha^−1^ yr^−1^).

**Figure 5:**
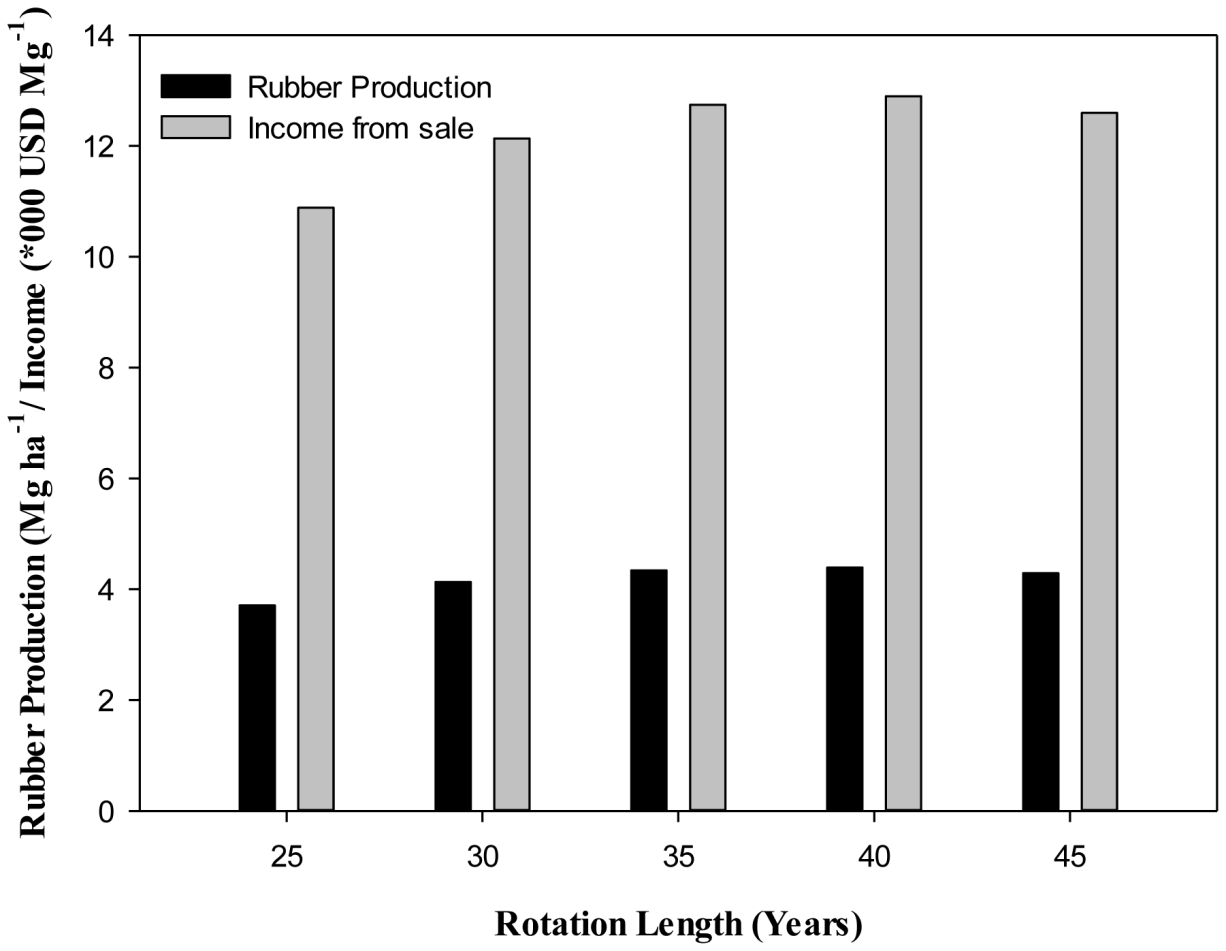
Comparison of income (*1000 USD Mg^−1^) and rubber production (Mg ha^−1^) at different rotation (1USD  = 6.13CYN).

The data received from the institute of land planning and environmental protection, farm management committee, Jinghong, Xishuangbanna revealed that based on the rubber production data from 8–43 years old plantation, the production of rubber is increased with the increase in years (Table 3). The regression analysis showed a polynomial (Fig. 4; R^2^ = 0. 81) relationship between age and rubber production. Using this regression model, production in 25, 30, 35, 40 and 45 years was determined. It was revealed that rubber production (Mg ha^−1^ yr^−1^) at 40 years was (4.39) maximum as compared to production at currently adopted rotation (35 years) (Fig. 5). To balance the economics and ecology, the total revenue/income that can be generated from the rubber production at different rotation lengths, the present (Oct, 2014) market price @ 2936.92USD Mg^−1^ was used. It was revealed that currently at 35 years rotation length the income from the rubber production is 12700 USD Mg^−1^ ha^−1^ that can be increased to 13000 USD Mg^−1^ ha^−1^ by changing the rotation up to 40 years (Fig. 5; 1USD  = 6.13 CYN).

**Table 3:** Rubber production (Mg ha^−1^ yr^−1^) and diameter at breast height (DBH) in rubber plantation of Xishuangbanna. China.

S.No	Age (Years)	DBH (cm)	Rubber Production (Mg ha^−1^ yr^−1^)
1	1–3	2.44	0
2	4–7	15.85	0
3	8–9	16.39	1.18
4	10–12	20.05	1.44
5	13–17	22.80	2.57
6	18–22	25.94	3.20
7	23–27	30.71	4.47
8	28–32	27.82	3.21
9	33–37	35.55	3.49
10	38–42	43.63	4.59
11	≥43	40.43	4.43

Source: Institute of land Planning and Environmental Protection, Farm Management Committee, Jinghong, Xishuangbanna.

## Discussion

### Reliability of the results

Reliability of the results of this study depends on, first how realistically CO_2_FIX model describes C cycling in forest and plantations and, second, the parameter values used. We evaluated the overall reliability of our results by comparing them to various studies carried out on carbon budgeting of the rubber plantations not only in Xishuangbanna but in other parts of the world (Table 4).

**Table 4:** Comparison of biomass, aboveground and soil C stocks in different studies of rubber plantations.

S.No	Reference*	Study area co-ordinates	Age	Aboveground Biomass (Mg ha^−1^)	Aboveground C Stock (MgC ha^−1^)	Soil C Stocks (MgC ha^−1^) (0–60 cm)
1	Present Study (2014)	21^o^ 41′N 101^o^ 25′E	40	229.58	114.79	55.18
2	Song and Zhang (2010) [37]	21^o^ 08′N 99^o^ 56′E	26	231.50	115.75	-
3	Jia et al., (2006)[38]	21^o^ 09′N 99^0^ 58′E	17	108.35	-	-
4	Castillo and Reyes (2004)[44]	14° 08′ N 12°12′ E	36	185.60	92.80	-
5	Huber et al., (2005)[45]	21^o^ 08′N 99^o^ 56′E	38	173.20	86.60	-
6	Cotta (2005) [46]	20^o^ 48′S 42^o^ 52′W	34	169.3	84.65	-
7	Song et al., (2013) [36]	21^o^ 55′N 101°15′E	49	-	122.89	-
8	de Ble'court et al., (2013) [28]	21^o^ 31′N 100^o^ 37′E	46	-	-	60.1
9	Wauters et al., (2008) [29]	48^o^ 55′ N 28^o^ 02′W	14	-	-	52.42
10	Wu et al., (2009) [47]	19^o^ 31′N 09^o^ 28′E	30	-	-	62.38
11	Sun (2013) [31]	21° 27′ N 100° 25′E	34	-	98.45	149.19 (at 1 m)

*Reference number link is in parenthesis.

The estimates of the soil carbon were found to be comparable to the literature reviewed. An average of 43.6 MgC ha^−1^ at a depth of 0.3–0.6 m in the rubber plantations of Menglong Township, Jinghong County of the Xishuangbanna prefecture, Yunnan, China, was reported by [28], whereas the present work revealed an average of 40.11 MgC ha^−1^ at the same depth for rubber plantation of Xishuangbanna using a CO_2_FIX model. Moreover, C stocks of 52.48 and 101.45 MgC ha^−1^ was reported for 0–60 cm depth at the age of 14 and 25years respectively in Ghana and Brazil [29]. In soil of mixed plantation of rubber and palm oil at the depth of 0–60 cm in SW Cameroon [30] presented 45 MgC ha^−1^. The soil carbon stocks at a depth of 1 m in rubber plantation of Xishuangbanna are 183.48, 113.58, 156.71, 133.78, 147.08 and 149.19 MgC ha^−1^ at the age of 3,7,9,21,27 and 34 respectively [31] (Table 4). In rubber plantation of Brazil for the depth of 0–50 cm, the average soil carbon is documented as 94.25 MgC ha^−1^ [32], [29].

### Effect of Rotation Length on Carbon Stocks at different age

The total C stocks at the end of the simulation period were higher than those at the end of first rotation due to accumulation of biomass and soil carbon with time. Determinants of C stocks include plantation age, the rate of volume, increment and wood density [33], [34].

Rubber plantations had highest CAI and wood density, thus the results are consistent with the findings of the authors who reported higher C stocks in stands with higher rates of diameter increments and wood densities [35], [36]. Similar results have been found in other studies, for instance 92, 106, 116, 122 and 140 Mg C ha^−1^ was reported by [36] at the age of 25, 30, 35, 40 and 45years in a rubber plantation using allometric equations and C flux data . In the present study C stocks and simulations were carried out at 25, 30, 35, 40 and 45years in order to find the most efficient rotation for rubber in light of Article 3.4 of the Kyoto protocol. In comparison, the biomass carbon reserves in the rubber plantation ecosystem of Xishuangbanna at the age of 3, 7, 9, 21, 27, 34 years were 2.79, 23.25, 38.65, 81.35, 87.53, 81.35 and 98.45 MgC ha^−1^ with an average of 55.34 MgC ha^−1^. With the development of the rubber tree, the average rate of C accumulation was reported as 2.08 MgC ha^−1^ yr^−1^ [31]. Fastest C accumulation rate 3.85 Mg C ha^−1^ yr^−1^ at 7–9 years old plantation, followed by the 3–7 year old (2.56 Mg C ha^−1^ yr^−1^) was reported by [37], which shows rapid growth of the rubber tree biomass carbon accumulation at early ages. From 9 to 27 years, the accumulation rate of rubber forest biomass carbon decreased, but, the accumulation rate of 27–34 years old rubber plantation is slightly higher than 21–27 years old plantation, but still below the average 3–34 year old (1.54 Mg C ha^−1^ yr^−1^) which lower than the average of the present study. At the age of seven year rubber, modelling of the tree biomass carbon accumulation [38] reported less value.

The four consecutive cycle regime allows for higher carbon stocks in all rotation ages because the shunt gap ensures that the replanted section reaches productive maturity before the next section is cut, and there is relatively less material available for fuel wood, decomposition and consequent emission of CO_2_ per unit time [33]. This growth in C stocks with rotation length is consistent with findings by [30], [39] and [40] by using the same simulation model. Through litter fall during the growth phase and higher inputs when the plantations are cut at the end of each rotation, soil C increases with time [41]. Such soil sequestration from biomass inputs is determined by the proportion of non-woody, fine and coarse litter fractions as well as rates of oxidation, decomposition and leaching. When C-rich biomass inputs are high and decompose slowly, the rates of leaching and oxidation are low, resulting in higher sequestration potential [42].

Our estimates about potential C sinks resulted from the elongated rotation lengths revealed that annual rate of carbon input (Mg C ha^−1^ yr^−1^) is highest at the rotation of 40 years (Fig. 2) because till 40 years of age, the rate of increment in the biomass of the tree was maximized. The rate of C input at the age of 45 gradually decreased due to gradual decrease in CAI of tree biomass. The increment in the tree biomass gradually increased from 25 to 40 years and then started declining, which affected the C inputs per year later on. So 40 years rotation age has been advocated as a larger carbon sink for rubber plantation from this study in light of Article 3.4 of the Kyoto Protocol. [31] reported the average rate of increase in C in rubber plantation is 2.08 which are lower than the computer model based estimation (4.2311 Mg ha^−1^ yr^−1^) of the present study.

Moreover, the economics of the rubber plantation pointed out that by enhancing the rotation would lead to more production as well as income from sales. The increase in rubber production with increasing age by planting certain clones has also been reported by [43].

## Conclusion

The investigation of the economics (income) of the rubber production and the ecology (C stocking) pointed out that an increase in both income and C stocks can be achieved by changing the rotation of rubber under the light of Article 3.4 of the Kyoto Protocol. In order to cope with the environmental hazards that may result in more erosion/runoff after the clear cutting at the age of 40 years, the introduction of some economically and ecologically important species should be carried out in between the rubber trees according to the suitability (mono culture or agroforestry) from the age of 35 years. These species may include *Coffea arabica, Theobroma cacao, Myristica yunnanensis, Bennettiodendron leprosipes, Gmelina arborea, Mesua ferrea, Erythrophleum fordii, Podocarpus fleuryi, Shorea chinensis, Dipterocarpus tubinatus.*

Moreover, comprehensive models that can pay attention to the variables which creates effects of high magnitudes of outputs should be develop with the incorporation of economics as well as ecological inputs and outputs. Future studies using these models should be conducted to serve as a guide to mitigate the risk to ecology on existing plantations and how the future expansions could incorporate the lessons learnt with the existing plantations. The loss of biodiversity and soil fertility should also be considered.
